# Feasibility of Mental Health Triage Call Priority Prediction Using Machine Learning

**DOI:** 10.3390/nursrep14040303

**Published:** 2024-12-20

**Authors:** Rajib Rana, Niall Higgins, Kazi Nazmul Haque, Kylie Burke, Kathryn Turner, Terry Stedman

**Affiliations:** 1 School of Mathematics, Physics and Computing, Springfield Campus, University of Southern Queensland, Springfield Education City, QLD 4300, Australia; rajib.rana@unisq.edu.au (R.R.);; 2 Mental Health and Specialist Services, West Moreton Health, Brisbane, QLD 4076, Australia; terry.stedman@health.qld.gov.au; 3 Metro North Mental Health, Metro North Health, Brisbane, QLD 4029, Australia; 4 School of Psychology, The University of Queensland, Brisbane, QLD 4072, Australia; 5 Australian Research Council’s Centre of Excellence for Children and Families over the Life Course, Brisbane, QLD 4068, Australia

**Keywords:** artificial intelligence, automated distress screen, deep learning, distress, mental health, spontaneous speech, triage, voice computing

## Abstract

Background: Optimum efficiency and responsiveness to callers of mental health helplines can only be achieved if call priority is accurately identified. Currently, call operators making a triage assessment rely heavily on their clinical judgment and experience. Due to the significant morbidity and mortality associated with mental illness, there is an urgent need to identify callers to helplines who have a high level of distress and need to be seen by a clinician who can offer interventions for treatment. This study delves into the potential of using machine learning (ML) to estimate call priority from the properties of the callers’ voices rather than evaluating the spoken words. Method: Phone callers’ speech is first isolated using existing APIs, then features or representations are extracted from the raw speech. These are then fed into a series of deep learning neural networks to classify priority level from the audio representation. Results: Development of a deep learning neural network architecture that instantly determines positive and negative levels in the input speech segments. A total of 459 call records from a mental health helpline were investigated. The final ML model achieved a balanced accuracy of 92% correct identification of both positive and negative instances of call priority. Conclusions: The priority level provides an estimate of voice quality in terms of positive or negative demeanor that can be simultaneously displayed using a web interface on a computer or smartphone.

## 1. Introduction

Mental illness is multifactorial, and understanding it requires not only measurable biometric data but also consideration of the complex social and historical context that impacts a person’s health outcomes [1]. Many psychiatric illnesses incorporate a voice component into their diagnostic criteria. For instance, disorganized speech is a key feature in diagnosing schizophrenia, while symptoms such as excessive talkativeness or pressure to keep talking characterize manic episodes in diseases such as Bipolar I and II [2]. Voice computing and machine learning (ML) hold significant promise for improving identification of those with high priority mental health needs by supporting clinicians in the assessment of callers’ need for care [3].

Mental health triage decision support aids can assist clinicians in responding appropriately to callers’ needs, and there is some evidence to indicate that they improve clinical judgment [4,5]. Contemporary clinical decision support systems for call center triage are mainly used to confirm clinical assessments and are now perceived as a support, rather than the previously-held views that they limit professional autonomy [6]. These systems typically are either knowledge-based, in which case medical questions and caller answers are used to make decisions according to guidelines, protocols, or clinical expertise, or data-driven, where statistical or machine learning models are used [7]. Currently, there is a pressing need to improve the clinical capability of health professionals, both mental health clinicians and others, in mental health call centers [8].

The most obvious obstacle in mental health telephone triage is the inability to use visual clues. This represents an integral part of decision-making that determines the care afforded to the caller. Imminent suicide risk is the most important triage issue. Clinicians are compelled to listen for signs of anxiety or depression even if the caller does not specifically state these symptoms. However, they are limited in their ability to communicate with the caller well enough for the clinician to make an assessment of priority for potentially life-threatening behavior on the part of the caller. In this paper, we investigate the feasibility of developing a call priority prediction system based on machine learning or deep learning to assist clinicians in identifying the priority of mental health needs for callers and supporting triage decision-making. This study proposes a novel approach that combines a variety of known sophisticated techniques in an attempt to support call takers’ efforts to manage this need on a daily basis.

The remainder of this paper is structured as follows: Section 2 reviews call priority through the use of machine learning; Section 3 describes the proposed approach for applying deep learning models in prediction and classification tasks, outlining the experimental design, dataset descriptions, and preprocessing approach; Section 4 presents the results of the prototype, highlighting its precision and clinical support capabilities; finally, Section 5 provides a discussion of the presented work, limitations, and conclusions.

## 2. Related Work

The application of machine learning in call center operations has gained significant attention in recent years. These techniques offer a data-driven approach that can address the limitations of traditional methods for identifying call priority. When applied to callers to a helpline, voice computing employs statistical learning using voice features as input to explain specific outputs related to symptoms of mental illness. This method requires a learning period, and relies on training datasets for success. For this literature review, a comprehensive search of the IEEE Xplore digital library was performed using the following terms: “call priority emergency services” and “call priority emergency services machine learning”.

The results show that relatively few studies have investigated the feasibility of call priority through machine learning, as demonstrated in Table 1. Most previous studies have used audio transcription, with only one [9] using audio and text separately. Given that speech is an important element of affective expression, this represents a notable gap in the literature regarding analysis of audio features to understand call priority. Most studies have used shallow machine learning methods as opposed to deep learning methods, again highlighting a significant gap in the literature related to the utility of advanced deep learning techniques for speech analysis. There is currently only one commercial product, Freshdesk, for analyzing speech to understand caller sentiment; however, it is applied solely to audio transcription. This limitation means that it does not benefit from analysis of affective information extracted through speech, which is key for conveying emotion and attitude.

### 2.1. Machine Learning in Call Centers

Most existing studies use call transcriptions to determine call priority. For example, Ceklic et al. [10] showed that employing natural language processing on emergency medical text has high predictive ability for distinguishing vehicle crashes that require the fastest ambulance response from those that do not. Similarly, Anthony et al. [11] presented an application of machine learning for classifying time-critical conditions, namely, sepsis, myocardial infarction and cardiac arrest, based on transcriptions of emergency calls from emergency services dispatch centers in South Africa. In [12], Defilippo et al. defined and implemented an AI-based module for managing patient emergency code assignments in emergency departments. Abi et al. [9] carried out similar research using both audio and text, demonstrating through their results that it is possible to use a selection of algorithms to predict whether a call will result in a serious injury based only on the caller’s speech. In their research, they were able to achieve accuracy of 71%. Two other studies used call records to estimate call priority. Rosa [13] et al. focused on identifying and classifying the emotional states of patients in real time, while Arngeir [14] addressed control design in human–AI collaboration so as to enhance rather than replace human decision-making processes.

**Table 1:** Summary of studies and products on call priority prediction using machine learning.

Study/ Product	Method	Accuracy	Dataset Used	Predicted Observation
Ceklic 2022 Perth, Western Australia [10]	Retrospective cohort studyNatural language processing on emergency medical text. Ensemble, K-nearest neighbors (k-NN), Naïve Bayes, Neural Network, Support Vector Machine	98%	Texts sent to paramedics en route to the traffic crash scene by the emergency medical dispatcher (EMD) in combination with dispatch codes for all traffic crashes attended by SJ-WA paramedics over the study period in the Perth metropolitan area.	Which incidents require a lights-and-sirens ambulance response.
Anthony 2021 South Africa [11]	Retrospective cohort studySupport Vector Machine (SVM), Logistic Regression, Random Forest, K-Nearest Neighbors (kNN).	95%	The dataset comprised 93 manually-transcribed emergency call conversations from various EMS contact centers in South Africa. The transcriptions were originally collected in various South African languages: English, Afrikaans, Zulu, and Sesotho.	Accurately identifying time-critical conditions, namely, sepsis, myocardial infarction, and cardiac arrest based off transcriptions of emergency calls.
Defilippo 2024 Italy [12]	Retrospective cohort studyGraph Neural Network	“High accuracy in predicting triage code.”	Publicly available historical data from the Kaggle platform containing relevant patient information such as vital signs, symptoms, and medical history.	Accurately identifying a triage code based on clinical presentation of a patient to an emergency department.
Abi 2023 France [9]	Retrospective cohort studyXGBoost, LSTM, TextCNN, XLM-RoBERTa, CamemBERT	71%	One week of emergency call recordings provided by the French SDIS 25 fireman station.	Predicting whether a call will result in a serious injury based only on the caller’s speech and assigning an appropriate priority to their call.
Freshdesk San Mateo, California, USA [15]	AI-driven Priority Assignment		Proprietary Customer Data	To assess the emotional tone of customer interactions.
Genesys Cloud Menlo Park, California, USA [16]	Predictive Routing with AI		Proprietary Customer Data	To set the priority of interactions while callers wait in a queue to ensure that high-priority calls are addressed promptly.
ZendeskSan Francisco, California, USA [17]	Machine Learning-Based Prioritization		Proprietary Customer Data	To prioritize customer calls while ensuring that high-priority issues are addressed promptly.

### 2.2. Commercially Available Products

Relatively few commercially available products employ machine learning and AI for call priority prediction. Freshdesk’s [15] sentiment analysis feature leverages AI to assess the emotional tone of customer interactions. Genesys Cloud [16] allows the call management system to set the priority of interactions using the “Set Priority” action within their Architect in-queue flows. Zendesk [17] can identify the priority of customer interactions using a combination of automation, AI, and custom configurations to enhance the efficiency and responsiveness of call centers.

Because relatively few studies that have been conducted in this domain and none of these are in the field of mental health, we drew from a range of state-of-the-art applications described in the existing literature that could contribute to identifying possible approaches for this study.

## 3. Methods

Phone callers’ speech is initially isolated using existing APIs, then features or representations are extracted from the raw speech and deep learning techniques are applied to determine the level of priority. Contrary to the traditional approach of employing audio features such as pitch and articulation rates, we instead use deep learning in an advanced machine learning technique for learning representations from audio data, then use this representation within our deep neural network to classify the call priority.

Our approach utilizes a sophisticated methodology to enhance the accuracy and efficiency of identifying and classifying caller speech. We developed a hierarchical classifier using deep learning neural networks. Specifically, we employed a machine learning model based on artificial neural networks, which we trained on a dataset consisting of 459 historical helpline calls. By iteratively adjusting the connections and weights within the network, we created an ML algorithm capable of recognizing patterns and making informed classification decisions.

The structure of the neural network comprises multiple processing layers, similar to neurons in the human brain, through which audio data are input. Each node within these layers performs simple calculations and transmits the results to the subsequent layers. Each layer independently focuses on distinct aspects or features of the raw speech data. These features are then fed into the hierarchical classifier, where the neural network makes its final decision based on the learned patterns and features in order to assign a priority level.

A loss function is used to calculate the difference between the actual and predicted classes. The model iteratively minimizes this discrepancy to achieve higher classification accuracy. Ultimately, we developed a prototype system integrating this classifier that assigns a priority level to the caller’s speech and displays the outcome through a browser-based interface.

A hierarchical classifier was designed using a grid search strategy, a common approach in which the algorithm iteratively selects the optimum solution at each decision point. This method ensures that the proposed classifier consistently achieves the best possible performance.

### 3.1. Dataset

We gathered call data from the mental health access line of a large public mental health hospital providing services to people with serious mental illness in Australia. This help line is a confidential mental health telephone triage service that acts as the first point of contact to public mental health services. This study was approved under the Public Health *Act* with ethical approval (project ID: 61948), and all calls were manually de-identified before audio analysis.

### 3.2. Preprocessing

Triage scores span a scale marked A to G, with A assigned to indicate the highest call priority and G the lowest call priority. Due to the small number of calls, we clustered them into high-priority and low-priority groups (see Table 2). We further split the call records into training and test sets, as shown in Table 3, with approximately 82% used for training and 18% for testing.

## 4. Results

We collected a total of 459 call records from the mental health access line. Of these callers, 198 were males and were 261 females, with a mean age of 39 years and 36 years, respectively. Table 4 describes these characteristics for each of the triage categories.

**Figure 1:**
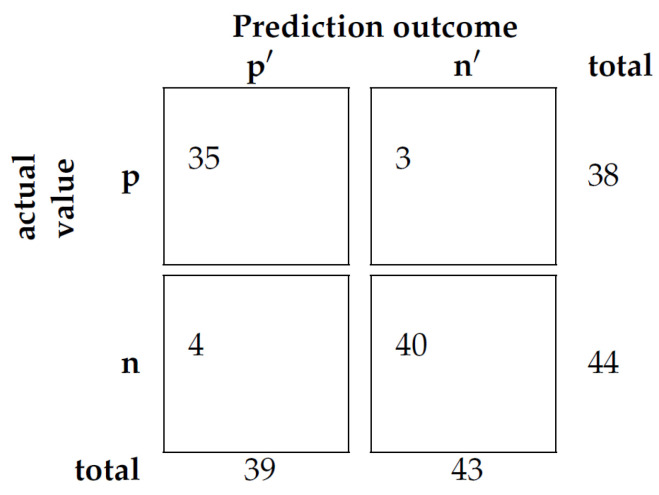
Confusion matrix.

The confusion matrix presented in Figure 1 provides an overview of the model’s performance. Out of 38 high-priority calls, 35 were accurately classified, while 3 were misclassified as low priority. Similarly, out of 44 low-priority calls, 40 were correctly classified, while 4 were misclassified as high priority. The model’s balanced accuracy, which measures its overall performance, was 92%, while its precision was 90%.

### Application

**Figure 2:**
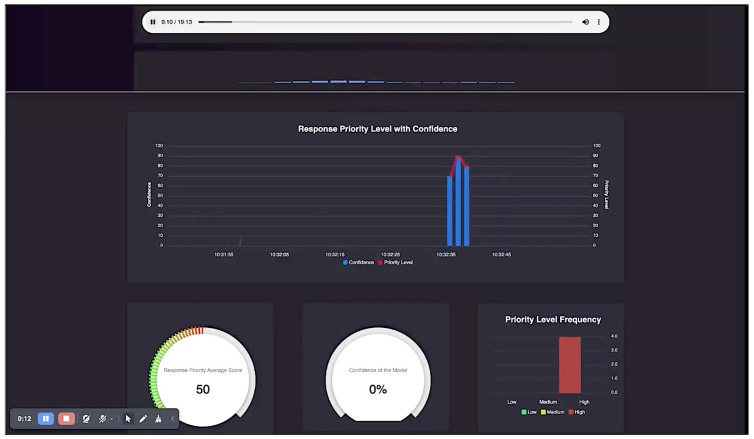
Application interface.

We developed an application that implements the algorithms proposed in this paper. An interface of the application is shown in Figure 2. This interface is displayed to the call taker as the call progresses. The very top block shows the audio being played. The next block shows the call priority assessed by the algorithm and the associated confidence in the assessment. The red line shows the priority level, and the corresponding blue bar shows the confidence of the algorithm in determining that priority level. The bottom level has two dials and a bar chart. The first dial shows the response priority average score. This is continually updated throughout the call, and can both increase and decrease. The next dial shows the average confidence. As soon as this dial reaches the value above a threshold (say, 90%), the priority level shown in the first dial can be used by the call taker as stable information from the algorithm even before the call finishes. However, the algorithm does not make the decision; the call taker eventually makes the decision based on the information provided by the algorithm. Finally, the third item is the histogram of priority levels, which shows how many of the audio chunks are high or low priority.

Each audio chunk is initially processed through a deep learning neural network to compute its representation. This representation is then fed into a hierarchical classifier, which determines the priority level and the corresponding confidence score for the priority inference. These priority levels are discrete, as a single priority is calculated for each audio chunk. For visualization purposes, in Figure 2 we have connected these discrete points to display them as a line.

## 5. Discussion

A significant challenge for clinicians when conducting telephone triage is the absence of visual cues. Observation of facial expressions, body language, and other behaviors are routine parts of face-to-face assessments in mental health. Without this crucial element, clinicians have an additional challenge in determining the care that should be provided to these callers. Clinicians must rely solely on auditory cues, even if callers do not explicitly mention symptoms such as anxiety or depression. It is essential for the clinician to effectively communicate with the caller in order to accurately assess the priority of potentially life-threatening behaviors.

The use of machine learning to assess the level of distress related to a caller’s mental health presents unique challenges. The effectiveness of the algorithms used in this context relies heavily on the quality of the triage categories used for model training. Therefore, we used the alternative approach of employing ML algorithms to predict functional consequences in the form of priority level of need rather than focusing solely on risk assessment. This justified our preprocessing approach of clustering the calls into high-priority and low-priority groups (illustrated in Table 2).

The prototype was developed using AI-based technology, and is intended to provide complementary support in streamlining tasks such as distress detection that do not require a ‘human touch’. The most beneficial function of AI in this study is to display important clinical indicators from the characteristics of the caller’s voice that may be unintentionally underestimated during the clinician’s risk assessment. This study demonstrates the feasibility of using machine learning and deep learning techniques to estimate call priority in mental health call centers. Much of the process for identifying meaningful patterns in callers’ voice data has clinical significance and is applicable to day-to-day practice. By leveraging nonlinear functions and avoiding preconceived assumptions, we were able to model complex relationships from a diverse dataset of over 450 different phone calls to a mental health help line. The use of multiple layers with interconnected nodes to process these data is the main novel difference between our deep learning approach and traditional methods. This technique enables our ML model to automatically learn hierarchical features from the raw speech data while at the same time reducing errors to produce a more accurate final assessment of call priority.

Our model achieved a balanced accuracy of 92%, indicating that it performs well across both sensitivity (true positive rate) and specificity (true negative rate) without favoring one over the other. This is particularly important because our training and testing sets were slightly imbalanced, and ideally would have been 80–20%. The precision outcome of 90% demonstrates that our model can correctly identify a high proportion of true positive predictions, with 90% of the instances identified as positive actually being positive. This is complemented by the high recall (or sensitivity) of 92%, as shown in Table 5. This result is crucial in mental health triage assessment, where false positives can result in potentially life-threatening outcomes for the caller. The F1 metric provides a single measure of our model’s performance. The high F1 score of 91.2% also indicates very good model performance, with both high precision and high recall. This result means that our model can accurately identify positive cases while also capturing most of the actual positive cases.

Capturing complex emotions through voice alone presents several challenges. We relied heavily on the retrospective data provided by clinical triage outcomes, as some related to suicidality were subtle, nuanced, and much harder to recognize; for example, quietly-spoken callers presented with greater risk of suicide more often than those with emotions such as anger that were easily identifiable using existing APIs. We did not have a usable volume of audio from callers with cultural differences, which would have been very valuable for understanding and interpreting culturally-specific clinical indicators of suicidal intention. Large global computer companies may have the ability to capture the necessary data from a wide range of cultures. One of the advantages of the approach taken for this study is that it is independent of the context and meaning of vocal cues such as tone of voice; thus, individual factors such as personality, gender, and level of mental health that can influence how emotions are conveyed and perceived had no influence on the computer-based assessment. This is the defining difference between human call takers and ML-based approaches that can make an impartial assessment of caller priority.

Addressing ethical considerations in machine learning and deep learning models is crucial. Future work should focus on developing frameworks to ensure that predictive models are fair, transparent, and accountable. It is essential that patients and clinicians have a clear understanding of the reasoning that AI-generated recommendations are based upon. For example, regular audits and adherence to ethical guidelines can help to mitigate biases and enhance trust in the system [18]. Clinicians and computer scientists must engage in discussions and debate with consumer representatives and policy-makers to help protect against potential abuse of the technology and allow for advancement of these technologies that could benefit so many in our society.

### Limitations

The study we conducted here examined whether the emotional features of a caller’s voice could be detected by machine learning algorithms to indicate the caller’s level of distress. The results provide an estimate of voice quality in terms of positive and negative demeanor. This was not provided to the listener in real time, and consequently did not influence the outcome of the call. Regarding affective value and ethical considerations, the assessment of AI solutions such as our prototype can be seen from two perspectives. On the one hand, AI approaches based on machine learning lack human ethics and reasoning, which limits their ability to address affective issues related to mental health. However, such methods are free from the biases that often influence human decisions. As long as our prototype remains confined to its role and does not substitute for mental health professionals, it can serve as an objective and unbiased tool to enhance decision-making.

Although the proposed model achieved high accuracy when interpreting human emotions from voice alone, we acknowledge that the full range of human emotions is still outstanding. Also, due to the inherent heterogeneity of mental illnesses, the triage category labels did not provide the specificity needed to achieve high sensitivity and specificity in our AI algorithms. This system has not yet been applied to different settings, and it is unknown whether it can be replicated in different populations.

Another possible limitation can be attributed to the sensitive nature of the data generated during mental health patient–clinician interactions and patient consultations. Additional challenges may also be attributed to the specific and sometimes complex diagnostic criteria defined in the DSM-5 for diagnosis of a psychiatric illness. Additionally, AI technologies require substantial amounts of data. This concern is not specific to the present study; indeed, the field of mental healthcare faces many limitations in accessing large high-quality datasets.

## 6. Conclusions

This research has explored the feasibility of implementing a deep learning-based call priority prediction system that performs an analysis of voice features rather than the actual content of what is said. The goal of the prototype ML application is to assist clinicians in identifying the mental health priority of callers and supporting their triage decisions.

Additionally, we aimed to address the false negative rate, as false positives are more acceptable when it comes to emergency services responses. In this context, false positives refer to low-priority calls being erroneously classified as high-priority, while false negatives indicate high-priority calls being mistakenly classified as low priority. Our false positive rate stands at 9%, which is low, but higher than the false negative rate of 8%.

Future work should focus on refining the model, exploring additional data sources, and addressing implementation challenges. A comprehensive approach involving continuous monitoring and iterative improvements will be essential for successful deployment. While voice computing holds great promise as a clinically assistive tool, its target clinical applications are yet to be fully defined. While our prototype has limitations in providing a true interpretation of a caller’s emotional state, it is crucial to appreciate that our primary goal is not to replace mental health clinicians; instead, our prototype serves as a supporting source of information for clinicians when they are focused on assessing callers experiencing mental distress. When used judiciously and within appropriate boundaries, it can effectively support mental health services in understanding and interpreting callers’ experiences without compromising equitable treatment.

## Figures and Tables

**Table 2:** Triage levels.

Categories	Triage Level
High Priority	A, B, C, D
Low Priority	E, F, G

**Table 3:** Distribution of training and testing samples.

	Train Samples	Test Samples
High Priority	171	38
Low Priority	206	44
Total	377	82

**Table 4:** Characteristics of all callers.

		**Triage Category (n = 459)**						
		**High Priority** **(n = 212)**				**Low Priority** **(n = 247)**		
		**A** **Emergency**	**B** **Very High Risk**	**C** **High Risk**	**D** **Mod Risk**	**E** **Low Risk**	**F** **Refer to GP**	**G** **Advice**
Male (n = 198)	Age Mean (SD)	41 (13.2)	37 (11.4)	39 (13.3)	38 (16.5)	35 (9.8)	44 (12.7)	38 (13.1)
	n (%)	15 (56%)	18 (58%)	25 (44%)	35 (36%)	57 (37%)	28 (52%)	20 (44%)
Female (n = 261)	Age Mean (SD)	33 (14.3)	32 (10.5)	33 (11.9)	35(14.5)	35(13.3)	38(16.2)	45(15.7)
	n (%)	12 (44%)	13 (42%)	33 (58%)	61 (63%)	90 (58%)	27(50%)	25(56%)
	**Total (n)**	27	31	58	96	147	55	45

**Table 5:** Overall performance results.

Metric type	Score %
True Negative Rate/Specificity	91%
True Positive Rate/Recall/ Sensitivity	92%
False Positive Rate	9%
False Negative Rate	8%
Balanced Accuracy	92%
Accuracy	91%
Precision	90%
F1 score	91.2%

## Data Availability

The data that support the findings of this study are not publicly available due to restrictions imposed by the Public Health Act 2005 (Queensland). Data contain sensitive information that could compromise the privacy of individuals. Requests for access to the data should be directed to PHA@health.qld.gov.au.

## Public Involvement Statement

There was no public involvement in any aspect of this research.

## Guidelines and Standards Statement

This manuscript was drafted against the TRIPOD+AI checklist for reporting of studies that develop a prediction model or evaluate its performance.
